# Survival analysis of localized prostate cancer with deep learning

**DOI:** 10.1038/s41598-022-22118-y

**Published:** 2022-10-24

**Authors:** Xin Dai, Ji Hwan Park, Shinjae Yoo, Nicholas D’Imperio, Benjamin H. McMahon, Christopher T. Rentsch, Janet P. Tate, Amy C. Justice

**Affiliations:** 1 Computational Science Initiative, Brookhaven National Laboratory, Upton, NY USA; 2 School of Computer Science, The University of Oklahoma, Norman, OK USA; 3 Theoretical Biology and Biophysics, Los Alamos National Laboratory, Los Alamos, NM USA; 4 VA Connecticut Healthcare System, West Haven, CT USA; 5 Schools of Medicine and Public Health, Yale University, New Haven, CT USA; 6 Department of Internal Medicine, Yale School of Medicine, New Haven, CT USA; 7 Faculty of Epidemiology and Population Health, London School of Hygiene & Tropical Medicine, London, UK

**Keywords:** Cancer, Computational biology and bioinformatics, Health care, Medical research, Oncology, Risk factors, Urology

## Abstract

In recent years, data-driven, deep-learning-based models have shown great promise in medical risk prediction. By utilizing the large-scale Electronic Health Record data found in the U.S. Department of Veterans Affairs, the largest integrated healthcare system in the United States, we have developed an automated, personalized risk prediction model to support the clinical decision-making process for localized prostate cancer patients. This method combines the representative power of deep learning and the analytical interpretability of parametric regression models and can implement both time-dependent and static input data. To collect a comprehensive evaluation of model performances, we calculate time-dependent C-statistics $C_{\text {td}}$ over 2-, 5-, and 10-year time horizons using either a composite outcome or prostate cancer mortality as the target event. The composite outcome combines the Prostate-Specific Antigen (PSA) test, metastasis, and prostate cancer mortality. Our longitudinal model Recurrent Deep Survival Machine (RDSM) achieved $C_{\text {td}}$ 0.85 (0.83), 0.80 (0.83), and 0.76 (0.81), while the cross-sectional model Deep Survival Machine (DSM) attained $C_{\text {td}}$ 0.85 (0.82), 0.80 (0.82), and 0.76 (0.79) for the 2-, 5-, and 10-year composite (mortality) outcomes, respectively. In addition to estimating the survival probability, our method can quantify the uncertainty associated with the prediction. The uncertainty scores show a consistent correlation with the prediction accuracy. We find PSA and prostate cancer stage information are the most important indicators in risk prediction. Our work demonstrates the utility of the data-driven machine learning model in prostate cancer risk prediction, which can play a critical role in the clinical decision system.

## Introduction

Prostate cancer is one of the most prevalent cancers among men in the United States. Approximately 12.5% of men will be diagnosed with prostate cancer, hereafter denoted as “PC”, during their lifetime^1^. In the United States, widespread prostate cancer screening leads to early diagnosis and medical intervention. However, treatment can incur severe side effects, e.g., incontinence or erectile dysfunction^2,3^. In those unlikely to benefit from treatment, it is especially critical to balance the trade-offs between different management options to maximize the quality of life and minimize unnecessary side effects. Localized disease (T1–4, N0, M0) accounts for 74.3% of prostate cancer diagnosis, and 5-year survival approaches 100%. On the other hand, prostate cancer still accounts for 5.6% of all cancer-related deaths. As such, a comprehensive risk estimation model for prostate cancer patients will be of great clinical value.

Thus far, there have been several related studies using different datasets and survival analysis methods^4–8^ for prostate cancer. The rapid advancement in machine learning techniques, particularly deep learning (DL), has made it possible to develop a personalized and automated risk prediction model to assist clinical decision-making^9^. One of the most significant obstacles in developing such a model is the lack of large-scale, high-quality Electronic Health Record, or EHR, data. As the largest integrated healthcare system in the United States, the Department of Veterans Affairs (VA) has collected more than 20 years of EHR data on 30 million veterans from multiple regions into the VA Corporate Data Warehouse (CDW). Via a collaboration between the VA and Department of Energy (DOE), we now have access to EHR and cancer registry data regarding more than 110,000 veterans diagnosed with localized prostate cancer. Our goal in this study is to take advantage of the large-scale, longitudinal, national EHR data from the VA and DOE’s high-performance computing power to develop a risk prediction model for localized prostate cancer patients using cutting-edge DL methods.

## Methods

### Population selection and data processing

To select localized prostate cancer patients, we used the American Joint Committee on Cancer (AJCC) TNM staging system^10^. “TNM” denotes the extent of the primary tumor (T) and whether cancer has spread to nearby lymph nodes (N) and distant parts of the body (M). For prostate cancer, the TNM staging system also considers the PSA level at the time of diagnosis and the Gleason score, which measures how likely the cancer is to grow and spread. By definition, for all stage I and II patients, the primary tumor is localized at the time of diagnosis.

Given the importance of the Prostate-Specific Antigen (PSA) test for prostate cancer, we collected all PSA test results, up to 10 years before diagnosis, for each patient. Other clinical input features include the Gleason score (range from 6-10) and the clinical prostate tumor stage, i.e., T-stage in the TNM staging system. Finally, the patients’ age and race information also were included. To sum up, the input data for our longitudinal model Recurrent Deep Survival Machine (RDSM) consisted of time-ordered PSA tests, age at each PSA test, and the time distance between each test and the time of diagnosis, as well as the Gleason score, T-stage, and patient race. For time-independent models, we replaced the PSA tests with their summary statistics, which included the number of PSA tests, maximum, minimum, average, last, and penultimate PSA values before diagnosis and a binary indicator showing if the last PSA test result was elevated compared with the penultimate test.

### Definition of patient outcomes and evaluation metrics

The relatively long survival time and low mortality rate of localized prostate cancer pose a great challenge in risk estimation. To get a more accurate disease prognosis evaluation over a shorter and practical timescale, we define a composite outcome as our event of interest:

- PSA > 50 ng/ml
- Metastatic diseases
- Prostate cancer mortality.

The event time is the earliest date of any of these three events. The censoring time is 1 year after the last PSA test. In cases where patients died of other causes before censoring, the censoring time instead is the time of death.

For better insight into the model performances over time, we calculate the time-dependent concordance-index $C_{\text {td}}(t)$^11^:

1 $$\begin{aligned} C_{\text {td}}(t)={\mathbb {P}}\left( {\hat{F}}\left( t \mid {\mathbf {x}}_{i}\right) >{\hat{F}}\left( t \mid {\mathbf {x}}_{j}\right) \mid \delta _{i}=1, T_{i}<T_{j}, T_{i} \le t\right) . \end{aligned}$$

Here, ${\hat{F}}\left( t \mid {\mathbf {x}}_{i}\right)$ is the cumulative distribution function (CDF) at time *t*, given input feature ${\mathbf {X}}$. To account for the high censoring ratio, we adjust $C_{\text {td}}(t)$ with the inverse probability of censoring weights^12^. Additionally, we test our models against the more conventional outcome, namely, prostate cancer mortality. In this study, we set the truncation time *t* to be 2, 5, and 10 years after diagnosis.

Depending if the input ${\mathbf {X}}$ is time-dependent, we employ two DL models, RDSM^13^ and Deep Survival Machine (DSM)^14^. As a benchmark, we also consider two popular machine learning models, Random Survival Forest (RSF)^15^ and Gradient Boosting Machine (GBM)^16^, along with the classical Cox model^17–20^. All three benchmark models are implemented using the scikit-survival package^21^.

$C_{\text {td}}(t)$^11^ is an useful metric for gauging model performance over time. By definition, it involves pairwise comparisons between different individuals. In the practical application, it is also helpful to compare the predicted survival probability with the actual survival status. In this regard, we consider Brier score^22^, which is defined as

2 $$\begin{aligned} \mathrm {BS}(t)=\frac{1}{n} \sum _{i=1}^{n} I\left( y_{i} \le t \wedge \delta _{i}=1\right) \frac{\left( 0-S\left( t \mid {\mathbf {x}}_{i}\right) \right) ^{2}}{{\hat{G}}\left( y_{i}\right) }+I\left( y_{i}>t\right) \frac{\left( 1-S\left( t \mid {\mathbf {x}}_{i}\right) \right) ^{2}}{{\hat{G}}(t)}, \end{aligned}$$

where $S\left( t \mid {\mathbf {x}}_{i}\right)$ is the predicted survival function with input *x*, $I(\cdot )$ is the indicator function, and $1/{\hat{G}}$ is an inverse probability of censoring weight estimated by the Kaplan-Meier estimator. Eq. (2) shows that the Brier score measures the distance between the predicted survival probability with the true survival status weighted by the censoring weights.

### Deep learning model overview

Our DL model is an extension to the traditional parametric regression model. The parametric model assumes the survival times, or the logarithm of the survival times, of the population follow a particular distribution. For example, the Weibull distribution is characterized by the shape parameter *k* and scale parameter $\lambda$. The corresponding hazard function takes the form $h(t) = \lambda k t^{k - 1}$, meaning that the risk can change over time depending on the value of *k*. Compared with the semi-parametric Cox model, the parametric model is free of the proportional hazards assumption, which may not be realistic in our case as the time to event is long and risk can vary over time.

**Figure 1:**
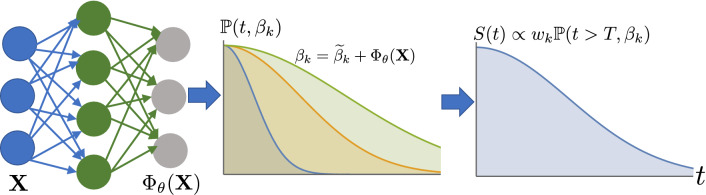
Deep learning model overview. The neural network is responsible for learning the feature representation $\Phi _{\theta }({\mathbf {X}})$, given the input ${\mathbf {X}}$. The parameters of all *k* distributions, $\beta _k$, and their mixing weights, $w_k$, are learned jointly during the training.

However, for the large-scale EHR data collected over a long period and across different locations, a single distribution function may be insufficient to characterize the whole population without introducing biases toward some specific subgroups. Moreover, we argue that a single distribution function contains too few parameters to utilize the rich information embedded in the large heterogeneous dataset. Thus, to increase the model capacity and reduce potential bias, we adapt a DL-augmented ensemble approach, which was first introduced in Ref.^13,14^. Figure 1 illustrates the schematics of our method’s pipeline. The primary idea is to model the conditional survival function ${\mathbb {S}}(t \mid X) \triangleq {\mathbb {P}}(T>t \mid X)$ as an *ensemble* of parametric distributions, and the parameters of each distribution function and mixing weights are learned from a neural network. This ensemble approach can help lower the variance and increase the out-of-sample performance, while the expressive power of DL models affords more efficient use of patients’ data.

### Ethical approval

DOE researchers worked under the approval of the VA Central IRB, per DOE/VA Memorandum of Understanding specific to the MVP-Champion collaboration. All methods were performed in accordance with the relevant guidelines and regulations. All participants provided written informed consent.

## Results

**Figure 2:**
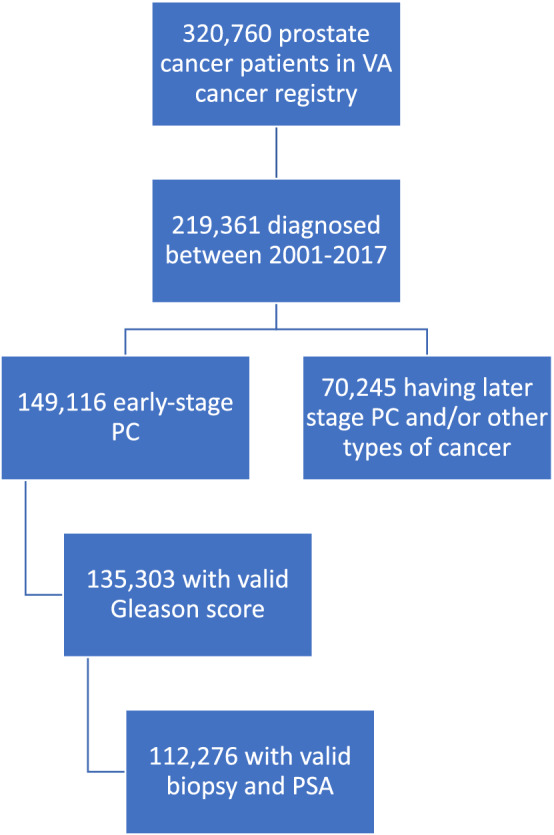
Patient selection flowchart. After gathering all prostate cancer patients diagnosed between 2001-2017 in the VA cancer registry, we have excluded patients satisfying any of the following criteria: (1) late-stage (metastasized) prostate cancer or having other types of cancer at the time of diagnosis, (2) no valid PSA test 1 year before diagnosis, (3) no valid Gleason score (Unknown or $<6$), and (4) no biopsy record for diagnosis.

**Figure 3:**
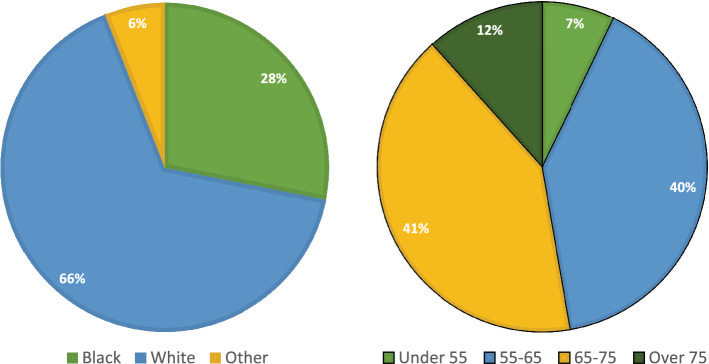
Distribution of race and age at diagnosis. The “Other” designation under race includes all Asian, Pacific Islander, Native American, and patients without valid race information.

Following the patient selection protocol outlined in Fig. 2, our study cohort comprised 112,276 localized prostate cancer patients. Before censoring, 7663 patients had the composite outcomes, and 3126 patients had PC-mortality outcome. The median age at the time of diagnosis was 65.5 years. The majority of the patients were either white (66%) or black (28%) (Fig. 3). Table 1 lists the clinical feature distributions of the entire cohort.

**Table 1:** Clinical feature distributions of the cohort.

Mean PSA (ng/ml)	8.83
Mean age at diagnosis	65.80
PSA counts	7.26
T-stage 1	70.28%
T-stage 2	27.96%
T-stage 3	1.59%
T-stage 4	0.17%
Gleason score 6	41.29%
Gleason score 7	42.32%
Gleason score 8	9.55%
Gleason score 9	6.31%
Gleason score 10	0.53%

Here, the mean PSA refers to the value of the last PSA test prior to diagnosis. PSA counts is the number of PSA tests up to 10 years before diagnosis. The Gleason score is the sum of primary and secondary scores.

We randomly split all patients into training (80%) and test set (20%) while ensuring the censoring ratio is consistent. Table 2 details the outcome statistics of the training and test sets. Because patients may experience multiple events before censoring, the number of composite outcomes is less than the sum of three single outcomes.

**Table 2:** Event statistics of different outcomes in the training and test sets.

Outcome	PSA > 50 ng/ml	Metastasis	PC-mortality	Composite outcome	Right-censored
Training set	4199	2410	2494	6130	83690 (93.17%)
Test set	1062	605	632	1533	20923 (93.17%)

**Figure 4:**
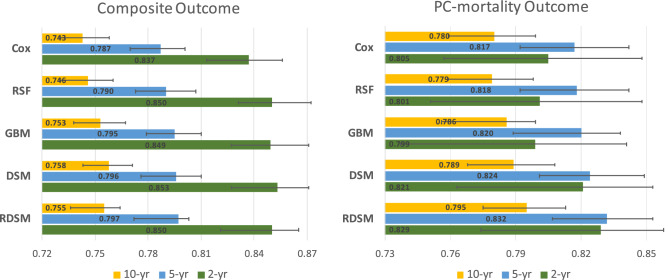
$C_{\text {td}}$ with 95% confidence interval (CI) for all tested models at near (2-year), mid (5-year) and long (10-year) time horizons for composite (left) and prostate cancer (PC)-mortality (right).

Figure 4 summarizes model performances on the test set at different event horizons for two outcomes. Across different time horizons and target outcomes, all of the machine learning models consistently outperformed the Cox model. For the composite outcome, the cross-sectional DL model DSM shows a slight advantage compared to other models, while for the PC-mortality outcome, the longitudinal model RDSM achieves the highest $C_{\text {td}}$ in all cases.

### Uncertainty quantification

**Figure 5:**
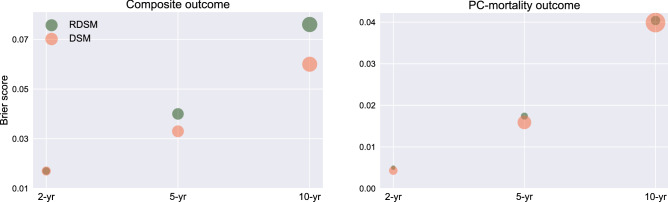
Brier scores for RDSM and DSM at different event horizons for composite (left) and PC-mortality (right) outcomes. Each dot size is proportional to the corresponding variance $v(t\mid X)$.

The significance of uncertainty quantification (UQ) is that it yields a meaningful metric about how confident the model is regarding the prediction. As $S(t \mid X)$ is the weighted average of parametric regression ensemble in our DL approach, we are able to calculate $v(t\mid X)$, the standard deviation of $S(t \mid X)$ for each prediction. Moreover, we can compare $v(t\mid X)$ with the Brier score to check if $v(t\mid X)$ is associated with the prediction accuracy. Per Fig. 5, for both RDSM and DSM, the correlations between the Brier scores and $v(t\mid X)$ are shown to be consistent, i.e., a lower variance indicates a lower Brier score. This result suggests that $v(t\mid X)$ is a reliable indicator for UQ, which is of great importance in real-world clinical decision-making.

### Subgroup analysis

**Figure 6:**
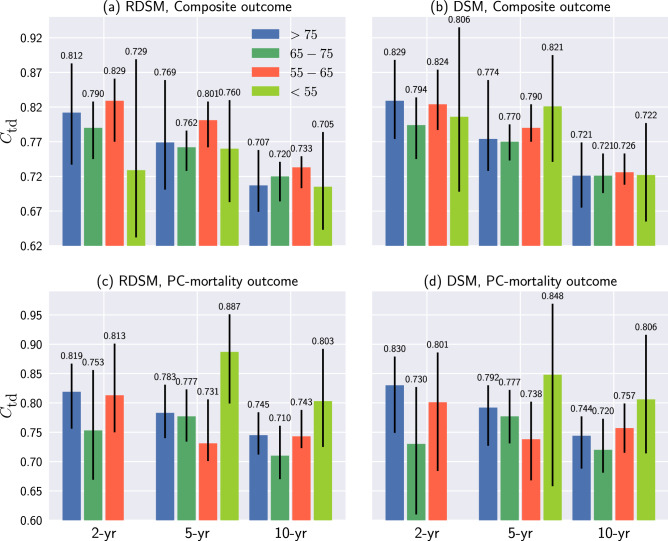
Age subgroup analysis for RDSM and DSM. Note that there is no incidence of PC-mortality in the $<55$ age group. Black lines denote 95% CI.

**Figure 7:**
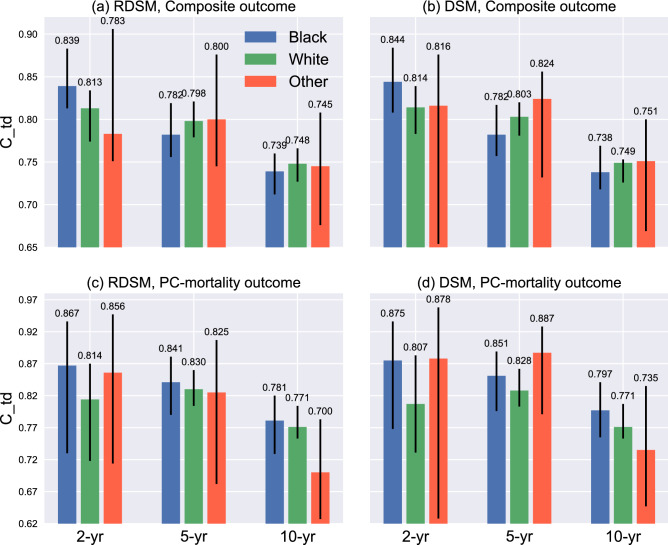
Race subgroup analysis for RDSM and DSM. Black lines denote 95% CI.

To study how our models perform in different race and age subgroups, we conduct subgroup analysis by excluding either race or age-related variables and testing models on each subgroup. Figures 6 and 7 show the results of RDSM and DSM for the age and race subgroups, respectively. Results from other models in each subgroup can be found in the Supplementary Information.

Compared with Fig. 4, both models show deteriorating performances across different age groups, especially for the long-term (10-year) prediction. A plausible reason is that the age specification in each subgroup leads to the reduced variance in the outcome, which, in turn, could have a negative impact on the $C_{\text {td}}$. Another explanation of performance drop is the shrinkage of patient numbers in each subgroup (Fig. 3). Empirically, DL models are susceptible to sample size reduction as they have more parameters to fit than traditional machine learning and statistical models. Yet, even for the two largest age groups, i.e., 55–65 and 65–75 years, the performance gaps still are significant. Conversely, the variances of $C_{\text {td}}$ among different race groups are smaller, particularly for the longer-term (5- and 10-year) predictions (Fig. 7). For example, RDSM achieves $C_{\text {td}}$ 0.84 and 0.78 for 5 and 10-year PC-mortality outcome prediction in the Black subgroup. Meanwhile, in the White subgroup, the corresponding numbers are 0.83 and 0.77, respectively. The insensitivity of $C_{\text {td}}$ regarding race indicates that it is not a useful indicator in predicting the PC prognosis for our models.

### Ablation study

Although our DL-based models achieve higher $C_{\text {td}}$ compared to the benchmark models, the black box nature of DL hinders the interpretability. To alleviate the problem, we conduct an ablation study to quantify how each input feature contributes to the risk estimation of DSM and RDSM.

We divide input features into four categories: PSA, age, race, and cancer stages. For the longitudinal model, we also include the time interval information. Our ablation approach drops each feature group and tracks model performances. However, bear in mind that neural networks in our DL models involve nonlinear interactions between each input. Hence, we remain cautious in interpreting the results.

**Table 3:** Ablation study results for the composite (left) and PC-mortality (right) outcomes.

(a) Composite outcome				(b) PC-mortality outcome			
Event horizon	2-yr	5-yr	10-yr	Event horizon	2-yr	5-yr	10-yr
Model				Model			
RDSM	0.850	0.797	0.755	RDSM	0.829	0.832	0.795
RDSM w/o interval	0.840	0.793	0.755	RDSM w/o interval	0.827	0.839	0.796
RDSM w/o race	0.848	0.787	0.750	RDSM w/o race	0.831	0.834	0.793
RDSM w/o age	0.845	0.789	0.744	RDSM w/o age	0.816	0.826	0.788
RDSM w/o stage	0.837	0.764	0.718	RDSM w/o stage	0.795	0.789	0.760
RDSM w/o psa	0.743	0.730	0.716	RDSM w/o psa	0.790	0.797	0.762
DSM	0.853	0.796	0.758	DSM	0.821	0.824	0.789
DSM w/o race	0.850	0.794	0.755	DSM w/o race	0.812	0.820	0.785
DSM w/o age	0.842	0.785	0.739	DSM w/o age	0.798	0.815	0.776
DSM w/o stage	0.831	0.762	0.718	DSM w/o stage	0.793	0.777	0.747
DSM w/o psa	0.736	0.731	0.717	DSM w/o psa	0.780	0.790	0.754

For reference, the results also include all input variables on the top row of the table.

Table 3 provides the ablation results for composite and PC-mortality outcomes and shows that race is the least important feature for both RDSM and DSM. The second least important feature is the age at diagnosis, especially for the composite outcome. This result is consistent with those of the subgroup analysis. In addition, we determine that the cancer stage information (Gleason score and T-stage) is crucial for the model performances, especially in the longer-term time horizons (5 and 10-year). Both RDSM and DSM experience huge performance drops without PSA-related features for the composite outcome. Naively, it is due to our definition of the composite outcome, which included a specific value of the PSA test (PSA > 50 ng/ml). Nevertheless, PSA-related features also have a significant influence on the model performances for the mortality outcome.

### Regional analysis

**Figure 8:**
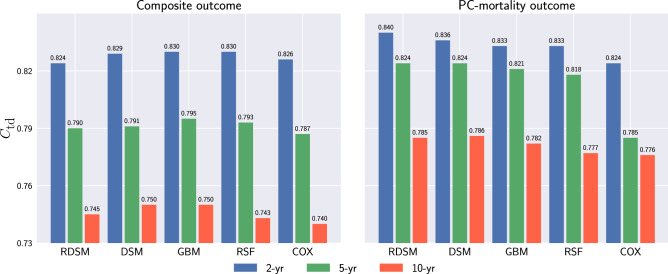
Averaged regional performance of all models.

One of the most important criteria to assess a model’s generalizability is independent external validation. As we had no such data at our disposal, we instead have devised a proxy method to approximate independent external validation. According to the definition of the United States Census Bureau, we divide all VA facilities into four main regions: Northeast, Midwest, South, and West. Each respective region accounts for 13%, 22%, 44%, and 19% of our cohorts (we have treated patients from other U.S. territories, which represented 2% of our total population, as a single group and put them into the training data). Then, we train our model using data from three regions and reserve the patients from the omitted region as the validation group. After getting the validation results from all regions, we show the results in Fig. 8 by doing a weighted average across all the regions, where the weight corresponds to the number of patients in each region. Supplementary Information features the results for the each individual region. Compared with Fig. 4, where patients from different regions are mixed, we find that all models generalized well geographically.

One noticeable exception occurred in the Midwest region (Table S2, Supplementary Information), where all models performed significantly worse than other regions for the 2-year composite outcome. We note the average PSA values are slightly higher (9.3 vs. 8.9 ng/ml) than the rest of the regions. While other important features (Gleason score; Clinical T) are quite similar. It is unlikely that the PSA difference is solely responsible for the anomalous results. As such, we leave the detailed investigation for future work.

## Discussion

In this study, we have developed and tested different survival models to predict localized prostate cancer prognosis in 2, 5, and 10-year time horizons using routinely available EHR data from the largest integrated healthcare system in the United States. In addition to the conventional PC-mortality outcome, we also have considered a composite outcome, which encompasses the PSA values, metastasis, and PC-mortality. Overall, in terms of the time-dependent concordance index $C_{\text {td}}$, two DL models, RDSM and DSM, outperform traditional machine learning models and the Cox model with a moderate margin (Fig 4). We also note that DSM and RDSM perform much faster than RSF and GBM in training and inference. In the short-term scenario (2-year), all models yield higher $C_{\text {td}}$ for the composite outcome than the PC-mortality outcome. We attribute this result to the low PC-mortality rate within the first 2-year horizon of diagnosis, resulting in a more skewed outcome distribution. For the longer period (5- and 10-year), all models experience considerable performance drop for the composite outcome, and the $C_{\text {td}}$ becomes lower than the corresponding PC-mortality prediction. The results suggest that our composite outcome, while being more clinically relevant for short-term prognosis management, poses greater challenges for achieving accurate long-term prediction.

For the PC-mortality outcome, our best model RDSM achieved the C-index 0.807 at the 10-year time horizon, which is comparable to other contemporary studies^5,7,8,23^ using multivariable approaches with similar input variables—although we clearly note that our results were not validated with external independent data. To compensate for this limitation, we have conducted a regional analysis by dividing the population into geographically distinct areas and using the omitted region as the test set. With one exception for the Midwest region, all models have demonstrated reasonable generalizability.

We also have performed analysis on subgroups stratified by age and race and found that the variances of $C_\text {td}$ among different age groups are higher than in race groups, suggesting that age is a more important predictor than race for $C_\text {td}$. We have conducted a thorough ablation study and identified that prostate cancer stage information and PSA-related features are the most important features for both composite and PC-mortality outcomes. Notably, the impact of prostate cancer stages (Gleason score and Clinical T) increases over time, while PSA-related features mainly impact the short-term (2-year) predictions. The ablation study also justifies the inclusion of PSA values in our definition of the composite outcome. Another interesting observation from the ablation study (Table 3) is that the longitudinal model RDSM did not benefit much from the time interval information. We conjecture this is because RDSM’s neural network architecture, Recurrent Neural Network (RNN), is a discrete sequential model that can be inefficient to learn continuous and irregularly spaced temporal signals without additional modification^24^.

One difference in our study with some previous work^5,6^ is we do not include treatment methods, e.g., hormone therapy and radiotherapy, as input variables in our models. Our focus is to provide an initial risk estimation before recommending any treatment.

Traditional statistical survival analysis can be roughly divided into three categories: non-parametric, semi-parametric, and parametric^25^. The semi-parametric Cox model and its variants have been widely adopted in the clinical survival analysis. The proportional hazards assumption of the Cox model often can be violated, especially when the event horizon is long. On the other hand, the parametric method, by assuming the survival times follow a particular distribution, is efficient and easy to interpret. However, its performance will suffer if the underlying distribution deviates significantly from the prior distribution. To overcome the limitation, we employ an ensemble approach by combining a large number of parametric regression models, where their combination weights and distribution parameters are learned via deep neural networks. The combination of neural network models and an analytical parametric approach enhances the model performance while preserving interpretability. As a natural extension of ensemble learning, our DL models can provide reliable UQ. We have shown that weighted variance of the predicted survival probability *S*(*t*) consistently correlates with the Brier score (Fig. 5). The reliable UQ will help clinicians make better-informed decisions.

The major strength of this study is that we are able to use a large cohort of localized prostate cancer patients from the VA national medical system, and we test the various survival models as the benchmark. Our DL models have demonstrated sufficient discriminating power along with useful UQ capability. Of note, the main limitation in our study is the lack of independent external validation. Without it, we acknowledge the inability to uncover the potential difference between veterans and the general population, which might impact the performance of real-world applications.

To conclude, our novel DL approach, equipped with UQ, can provide accurate, individualized risk estimation for localized prostate cancer patients. This study may further motivate the implementation of a clinical decision system augmented by artificial intelligence for prostate cancer prognosis management.

## Supplementary Information

Supplementary Information.

## Data Availability

The data used in this study cannot be made available due to restrictions related to the use of EHR data.

## Code availability

The code used in this study is available from the corresponding author (X.D., xdai@bnl.gov) upon request.
