# Supplementary material for: Survival analysis of localized prostate cancer with deep learning
Source: Sci Rep. 2022 Oct 24;12:17821. doi: 10.1038/s41598-022-22118-y (PMC9592586; doi:10.1038/s41598-022-22118-y)
Supplement: Supplementary file 1 — Supplementary Information. [file 41598_2022_22118_MOESM1_ESM.pdf]

Supplementary Information

The supplementary information contains the mathematical and training details of the models studied in the main text, and detailed results of regional and subgroup analysis.

Deep learning model & training details

The first step to build DSM or RDSM is choosing a distribution function $\mathbb{P}(t,\beta)$ for the parametric models, preferably the Log-Normal or Weibull distribution, because their closed form of CDF can simplify the gradient descent optimization. Here we use β to denote all the relevant parameters of the distribution function. Then we initialize k such distributions with some random prior parameters $\tilde{\beta}_k$. The input features x_i of patient i, are passed through the neural network Φ_θ to find the representation $\Phi_\theta(x_i)$.

More precisely, we have

$$\beta_k = \tilde{\beta}_k + \Phi_\theta(x_i) \tag{1}$$

The mixture weights w_k are also learned jointly via the optimization procedure outlined below. Depending on whether the input data is time-dependent or not, Φ_θ can be either a Recurrent Neural Network (RNN) or a Multi-Layer Perceptron (MLP), and we call them Recurrent Deep Survival Machine (RDSM) and Deep Survival Machine (DSM), respectively. In practice, we choose either Long short-term memory (LSTM) [1] or Gated recurrent unit (GRU) [2] as the concrete realization of the RNN module in the RDSM. The training proceeds by calculating the maximum likelihood estimator, which amounts to minimizing the following loss function:

$$\mathscr{L}_{\text{combined}} = \mathbf{ELBO}_U(\Theta) + \alpha \cdot \mathbf{ELBO}_C(\Theta) \tag{2}$$

where the first term denotes the uncensored loss,

$$\mathbf{ELBO}_U = \sum_{i=1}^{|\mathscr{D}|} \left(\underset{Z\sim(\cdot|\mathbf{x}_i,w)}{\mathbb{E}} [\ln \mathbb{P}(T = t_i \mid Z, \beta_k,)] \right), \tag{3}$$

and the second term is for the censored loss,

$$\mathbf{ELBO}_C = \sum_{i=1}^{|\mathscr{D}|} \left(\underset{Z\sim(\cdot|\mathbf{x}_i,w)}{\mathbb{E}} [\ln \mathbb{P}(T > t_i \mid Z, \beta_k)] \right). \tag{4}$$

Here $\alpha \in [0,1]$ is a discount factor and we treat it as a hyperparameter, and w denotes the mixture weight. To mitigate the long-tail bias, we add L_2 regularization for β_k during the training. The final survival probability $S(t|X)$ is the weighted average over k distributions:

$$S(t \mid X) = \sum_w w_k \mathbb{P}_k(T > t \mid X, \beta_k) \tag{5}$$

Hyper-parameter tuning & Model structure

We use 15% of the randomly selected training data as the validation set and performed hyper-parameter optimization for two deep learning models DSM and RDSM. The results reported in Fig. 3 of the main text are obtained from the test set using the models having the lowest loss on the validation set. We adopt a similar strategy for optimizing two traditional machine learning models, GBM and RSF, and select model parameters with the highest C_{td} on the validation set. Whereas for the Cox model, we perform 5-fold cross-validation on the entire training set and report the result on the test set using the parameter set having the highest average C_{td} in the cross-validation. To maximize the performance, we perform separate hyper-parameter tuning against two outcomes, i.e., composite and PC-mortality for each model.

The detailed DL model structure is as follows. For the composite outcome, we use a 2-layer LSTM model with 64 hidden units in each layer as the neural network module for RDSM, and initialize 40 parametric regression models with the LogNormal distribution, while we use a 2-layer MLP with 32 hidden units per layer for DSM, the number of parametric regression model is 90, which follows the Weibull distribution.

For the PC-mortality outcome, the only difference in RDSM is the change of learning rate from 0.03 to 0.0075. For DSM, the number of parametric regression model becomes 60, and the learning rate changes from 0.002 to 0.0005. We implement our DL models using PyTorch and use the Adam optimizer with a batch size of 2048 during training.

It is worth mentioning that we use the same parameters in the regional and subgroup analysis as the general case to ensure a fair comparison. We expect that if we perform hyper-parameter tuning in each case, we could see moderate performance increases for all models. Two traditional machine learning models, GBM and RSF are computationally expensive thus forbidding extensive hyper-parameter tuning. For example, training a RSF model on the whole training set can take more than 4 days, while RDSM and DSM only require a few minutes (60 epochs).

Regional Study Results

Table S2, S3 and S4 show the validation results using patients from South, Midwest and West regions.

(a) Composite outcome

Event Horizon	2-yr	5-yr	10-yr
**Model**			
RDSM	**0.862**	**0.824**	**0.765**
DSM	0.856	0.822	**0.765**
GBM	0.856	0.822	0.761
RSF	0.853	0.818	0.756
Cox	0.858	0.819	0.753

(b) PC-mortality outcome

Event Horizon	2-yr	5-yr	10-yr
**Model**			
RDSM	**0.881**	**0.863**	**0.807**
DSM	0.870	0.844	0.799
GBM	0.847	0.843	0.792
RSF	0.850	0.841	0.789
Cox	0.855	0.844	0.790

Table S1. Validation results for the composite (left) and PC-mortality (right) outcome using the Northeast cohort.

(a) Composite outcome

Event Horizon	2-yr	5-yr	10-yr
**Model**			
RDSM	0.824	0.788	0.742
DSM	0.836	0.795	0.750
GBM	0.838	**0.796**	**0.752**
RSF	**0.840**	**0.796**	0.750
Cox	0.833	0.791	0.745

(b) PC-mortality outcome

Event Horizon	2-yr	5-yr	10-yr
**Model**			
RDSM	0.813	0.805	0.772
DSM	0.823	**0.815**	**0.779**
GBM	0.827	0.812	0.777
RSF	**0.828**	0.812	0.773
Cox	0.817	0.809	0.774

Table S2. Validation results for the composite (left) and PC-mortality (right) outcome using the South cohort.

Subgroup Analysis Results

The stratification of different age and race subgroups can be found in Fig. 2 of the main text. Table S5, S6, S8 show the result for three different age groups. Table S9 and S11 show the results for the white and the other race groups.

(a) Composite outcome

Event Horizon	2-yr	5-yr	10-yr
**Model**			
RDSM	0.761	**0.764**	0.737
DSM	0.756	0.763	**0.740**
GBM	0.760	0.763	0.736
RSF	**0.764**	0.762	0.723
Cox	**0.764**	0.754	0.724

(b) PC-mortality outcome

Event Horizon	2-yr	5-yr	10-yr
**Model**			
RDSM	**0.848**	**0.838**	**0.798**
DSM	0.830	0.826	0.792
GBM	0.817	0.824	0.789
RSF	0.813	0.817	0.786
Cox	0.823	0.817	0.781

Table S3. Validation results for the composite (left) and PC-mortality (right) outcome using the Midwest cohort.

(a) Composite outcome

Event Horizon	2-yr	5-yr	10-yr
**Model**			
RDSM	0.875	0.803	0.747
DSM	**0.882**	0.809	0.750
GBM	0.878	**0.810**	**0.752**
RSF	0.878	0.805	0.743
Cox	0.863	0.797	0.737

(b) PC-mortality outcome

Event Horizon	2-yr	5-yr	10-yr
**Model**			
RDSM	**0.865**	**0.827**	**0.784**
DSM	0.852	**0.827**	**0.784**
GBM	0.857	0.821	0.777
RSF	0.855	0.817	0.767
Cox	0.820	0.810	0.773

Table S4. Validation results for the composite (left) and PC-mortality (right) outcome using the West cohort.

Event Horizon	2-yr	5-yr	10-yr
**Model**			
RDSM	0.729	0.760	0.705
DSM	0.806	0.821	0.722
GBM	0.826	0.813	0.713
RSF	**0.837**	**0.827**	**0.725**
Cox	0.812	0.828	0.719

Event Horizon	2-yr	5-yr	10-yr
**Model**			
RDSM	N/A	**0.887**	0.803
DSM	N/A	0.848	0.806
GBM	N/A	0.794	0.773
RSF	N/A	0.803	0.800
Cox	N/A	0.863	0.816

Table S5. Age Subgroup (age < 55) analysis for the composite (left) and PC-mortality (right) outcome. The 2-yr C-index is not applicable for the PC-mortality as all subjects in this age group are censored.

(a) Composite outcome

Event Horizon	2-yr	5-yr	10-yr
**Model**			
RDSM	0.829	0.801	0.733
DSM	0.824	0.790	0.726
GBM	0.829	**0.806**	**0.740**
RSF	**0.839**	0.806	0.739
Cox	0.830	0.796	0.729

(b) PC-mortality outcome

Event Horizon	2-yr	5-yr	10-yr
**Model**			
RDSM	**0.813**	0.731	0.743
DSM	0.801	0.738	0.757
GBM	0.785	0.734	**0.759**
RSF	0.786	0.746	0.759
Cox	0.779	0.738	0.752

Table S6. Age Subgroup (age 55-65) analysis for the composite (left) and PC-mortality (right) outcome.

(a) Composite outcome

Event Horizon	2-yr	5-yr	10-yr
**Model**			
RDSM	0.790	0.762	0.720
DSM	0.794	**0.770**	0.721
GBM	0.796	0.766	0.721
RSF	0.798	0.766	0.713
Cox	0.785	0.756	0.709

(b) PC-mortality outcome

Event Horizon	2-yr	5-yr	10-yr
**Model**			
RDSM	**0.753**	**0.777**	0.710
DSM	0.730	0.777	**0.720**
GBM	0.751	0.775	0.711
RSF	0.737	0.762	0.712
Cox	0.727	0.765	0.706

Table S7. Age Subgroup (age 65-75) analysis for the composite (left) and PC-mortality (right) outcome.

(a) Composite outcome

Event Horizon	2-yr	5-yr	10-yr
**Model**			
RDSM	0.812	0.769	0.707
DSM	0.829	0.774	0.721
GBM	0.820	**0.775**	0.722
RSF	0.823	0.772	0.719
Cox	0.822	0.762	0.713

(b) PC-mortality outcome

Event Horizon	2-yr	5-yr	10-yr
**Model**			
RDSM	**0.819**	0.783	0.745
DSM	0.830	0.792	0.744
GBM	0.802	**0.791**	0.746
RSF	0.813	0.783	0.739
Cox	0.797	0.773	0.734

Table S8. Age Subgroup (age $>$ 75) analysis for the composite (left) and PC-mortality (right) outcome.

(a) Composite outcome

Event Horizon	2-yr	5-yr	10-yr
**Model**			
RDSM	**0.833**	0.796	**0.760**
DSM	0.814	0.803	0.749
GBM	0.815	**0.806**	0.751
RSF	0.820	**0.806**	0.751
Cox	0.799	0.788	0.733

(b) PC-mortality outcome

Event Horizon	2-yr	5-yr	10-yr
**Model**			
RDSM	**0.814**	0.830	**0.781**
DSM	0.807	0.828	0.771
GBM	0.802	0.831	0.776
RSF	0.820	**0.834**	0.773
Cox	0.789	0.831	0.771

Table S9. Race Subgroup (white) analysis for the composite (left) and PC-mortality (right) outcome.

References

1. Hochreiter, S. & Schmidhuber, J. Long short-term memory. *Neural computation* **9**, 1735–1780 (1997).
2. Cho, K. *et al.* Learning phrase representations using rnn encoder-decoder for statistical machine translation. *arXiv preprint arXiv:1406.1078* (2014).

(a) Composite outcome

**Event Horizon**	2-yr	5-yr	10-yr
**Model**			
RDSM	0.839	0.782	0.739
DSM	0.844	0.782	0.738
GBM	0.847	0.790	**0.743**
RSF	**0.851**	**0.792**	0.740
Cox	0.839	0.769	0.730

(b) PC-mortality outcome

**Event Horizon**	2-yr	5-yr	10-yr
**Model**			
RDSM	0.867	0.841	0.781
DSM	**0.875**	**0.851**	0.797
GBM	0.845	0.834	0.790
RSF	0.843	0.835	0.797
Cox	0.874	0.846	**0.808**

Table S10. Race Subgroup (black) analysis for the composite (left) and PC-mortality (right) outcome.

(a) Composite outcome

**Event Horizon**	2-yr	5-yr	10-yr
**Model**			
RDSM	0.773	0.800	0.745
DSM	0.816	**0.824**	**0.751**
GBM	0.793	0.801	0.741
RSF	**0.826**	0.813	0.750
Cox	0.807	0.799	0.749

(b) PC-mortality outcome

**Event Horizon**	2-yr	5-yr	10-yr
**Model**			
RDSM	0.856	0.825	0.700
DSM	0.878	**0.887**	**0.735**
GBM	0.863	0.834	0.709
RSF	**0.897**	0.803	0.672
Cox	0.883	0.876	0.734

Table S11. Race Subgroup (other) analysis for the composite (left) and PC-mortality (right) outcome.
